# Early Favourable Outcomes of Valve Repair in Congenital Heart Surgery

**DOI:** 10.1093/icvts/ivaf273

**Published:** 2025-11-26

**Authors:** Muhammed Ikbal Aydin, Eiri Kisamori, Mitchell Haverty, Rittal Mehta, Aybala Tongut, Manan Desai, Gerard Martin, Wayne Jay Franklin, Yves d’Udekem

**Affiliations:** Department of Cardiovascular Surgery, Children’s National Hospital, Washington, DC, 20010, United States; Pediatric Cardiac Surgery, Columbia University Medical Center, New York, NY, 10032, United States; Department of Cardiovascular Surgery, Children’s National Hospital, Washington, DC, 20010, United States; Department of Cardiovascular Surgery, Children’s National Hospital, Washington, DC, 20010, United States; Department of Cardiovascular Surgery, Children’s National Hospital, Washington, DC, 20010, United States; Department of Cardiovascular Surgery, Children’s National Hospital, Washington, DC, 20010, United States; Department of Cardiology, Children’s National Hospital, Washington, DC, 20010, United States; Department of Cardiology, Children’s National Hospital, Washington, DC, 20010, United States; Department of Cardiovascular Surgery, Children’s National Hospital, Washington, DC, 20010, United States

**Keywords:** congenital valve repair, semilunar valve, atrioventricular valve

## Abstract

**Objectives:**

Mechanical valve replacement is often used as the therapeutic option in valvular heart surgery in children. Evidence suggests that this should change to provide optimal long-term survival for this growing population. We reviewed our current practice in valve repair in congenital heart disease and analysed its outcomes.

**Methods:**

A total of 90 patients (30 semilunar valve and 60 atrioventricular [AV] valve) underwent valve repair between September 2020 and December 2024. Operative data and follow-up information were gathered retrospectively. Kaplan-Meier calculations were used for survival and freedom from reoperation analysis. Cox regression analysis was used to assess risk factors for mortality (single ventricle physiology, age and weight at time of surgery, bicuspid aortic valve, and complexity of repair). Complexity of repair was defined as the application of 3 or more repair techniques.

**Results:**

Estimated survival at 12 months for semilunar and AV valve groups was 95.2% and 95%, respectively. Estimated freedom from reoperation at 12 months for repair of semilunar and AV valves was 95.8% and 95.6%, respectively. Single ventricle morphology (HR [hazard ratio], 5.2; 95% confidence interval [CI], 1.3-20.8; *P* = .0198) and younger age at time of surgery (HR, 0.7; 95% CI, 0.6-0.9; *P* = .0223) were associated with increased risk of mortality.

**Conclusions:**

Valve repair in congenital heart disease provides reliable early outcomes in this complex population. The worst outcomes are expected in patients with single ventricle requiring surgery in early life.

## Graphical abstract

**Figure:**
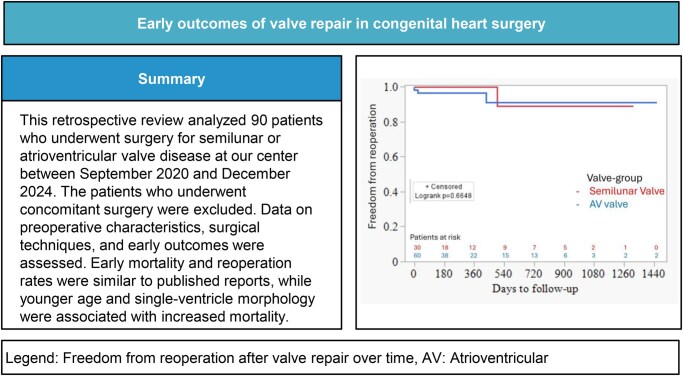

## INTRODUCTION

The scope of valve repair in congenital cardiac surgery has evolved in the last 2 decades.^1^^,^^2^ It was initially limited to primary congenital lesions of the mitral and tricuspid valve apparatus and limited to blade commissurotomy of aortic and pulmonary valve lesions. Mechanical valve replacement has been a cornerstone of our valvular heart surgery practice, but this trend may be changing. With the ever-increasing survival of our population of patients with congenital heart disease, our practice in valve repair in the field of congenital heart disease has evolved.^3^^,^^4^

We intended to review our current practice in valve repair in congenital heart disease to identify its current scope, its results, and limitations.

## METHODS

The design of the study was approved by our Institutional Review Board and the need for consent was waived because of retrospective nature of study (Pro00015566). In accordance with The WMA Declaration of Taipei, the Children’s National Institutional Review Board approved the establishment of and monitors the use of the research database (IRB Pro00015566, July, 2021). Data were obtained from medical and electronic patient records. Between September 2020 and December 31, 2024, a total of 1319 cardiopulmonary bypass (CPB) procedures were performed; 279 patients underwent valve surgery. Out of the 279, 90 individuals were identified to have undergone surgery with primary indication of moderate or severe valve disease. The duplicate patients and those who had valve repair secondary to intracardiac defects were not included. Over the same time period, there were 69 primary valve replacements, 34 redo valve replacements as well as 32 Ross operations.

Valve repairs were performed on systemic semilunar valves in 30 patients and on atrioventricular (AV) valve in 60 patients. Eight patients underwent both semilunar valve repair and AV valve repair. For these 8 patients, those who received a more complex semilunar valve repair were placed in the semilunar valve repair cohort, while those who underwent a more complex AV valve repair were placed in the AV valve repair cohort for statistical analysis. A proportion of patients in each group had previous cardiac procedures which included previous cardiac surgeries and catheter interventions. We described applying 3 or more repair techniques on a valve as complex repair. Outcomes of interest were early and late mortality and early and late reoperation. Early mortality and early reoperation were defined as occurring within 30 days after surgery. Late mortality and late reoperation were defined as occurring more than 30 days after surgery. Follow-up was obtained through chart review of outpatient clinic records, echocardiography, and subsequent procedures notes from operating room and catheter laboratory. Follow-up was concluded December 31, 2024.

### Statistical analysis

Data were retrospectively analysed and presented as absolute numbers and percentages for categorical variables. Continuous variables were first evaluated for distributional assumptions using normality tests (Shapiro-Wilk) and are reported as means with standard deviations or medians with interquartile ranges (IQRs), as appropriate. Survival distributions were estimated using Kaplan-Meier curves. Group comparisons of survival and freedom-from-reoperation were performed using the log-rank test, with *P*-values derived from these comparisons displayed alongside the Kaplan-Meier curves. Individual predictors were first examined using bivariable Cox regression models, in which the outcome and one predictor variable were included at a time. All the assumptions were assessed. Model performance and calibration were evaluated using standard diagnostics. A 2-sided *P*-value < .05 was considered statistically significant. All variables included in the analysis were complete, with no missing data identified after verification against electronic medical and operative records. Accordingly, multiple imputations were not required, and complete-case analysis was equivalent to the full dataset. The statistical analysis was performed using SAS 9.4 software (SAS, Cary, NC, USA).

## RESULTS

The demographic characteristics of the 90 patients of the study are outlined in **Table 1**. Surgical interventions spanned a wide age range, from 1 day old to 44 years.

**Table 1.** Patient Characteristics

Variable	Total (*n* = 90)	Semilunar valve (*n* = 30)	Atrioventricular valve (*n* = 60)
Sex, male, *n* (%)	43 (47.7)	19 (63.3)	24 (40)
Age, year (IQR)	5.4 (1.4-13.5)	13 (6.9-16.7)	3.9 (0.9-9.4)
0-1 month, *n* (%)	4 (4.4)	4 (13.3)	0
1-12 months, *n* (%)	16 (17.7)	1 (3.3)	15 (25)
1-5 years, *n* (%)	21 (23.3)	2 (6.6)	19 (31.6)
5-10 years, *n* (%)	16 (17.7)	4 (13.3)	12 (20)
>10 years, *n* (%)	33 (36.6)	19 (63.3)	14 (23.3)
Weight, kg (IQR)	16.8 (9-44.2)	41.7 (17.1-65.4)	14 (8-22.1)
<2.5 kg, *n* (%)	0	0	0
2.5-3.5 kg, *n* (%)	4 (4.4)	4 (13.3)	0
3.5-5 kg, *n* (%)	9 (10)	1 (3.3)	8 (13.3)
5-10 kg, *n* (%)	10 (11.1)	0	10 (16.7.3)
>10 kg, *n* (%)	67 (74.4)	25 (83.3)	42 (70)
Previous cardiac procedures, *n* (%)	57 (63.3)	9 (30)	48 (80)
Previous cardiac operations, *n* (%)	15 (16.6)	4 (13.3)	11 (18.3)
Regurgitation, *n* (%)	75 (83.3)	26 (86.6)	49 (81.6)
Stenosis, *n* (%)	15 (16.7)	4 (13.3)	11 (18.3)
Single ventricle, *n* (%)	23 (25.5)	0	23 (38.3)
Unicuspid valve, *n* (%)	1 (1.1)	1 (3.3)	0
Bicuspid valve, *n* (%)	12 (13.3)	12 (40)	0
Truncus valve, *n* (%)	5 (5.5)	5 (16.6)	0
Marfan syndrome, *n* (%)	2 (2.2)	2 (6.6)	0
Common atrioventricular valve repair, *n* (%)	7 (7.7)	0	7 (11.6)
Left atrioventricular valve repair, *n* (%)	16 (17.7)	0	16 (26.6)
Right atrioventricular valve repair, *n* (%)	5 5.5)	0	5 (8.3)
Mitral valve repair, *n* (%)	15 (16.6)	0	15 (25)
Tricuspid valve repair, *n* (%)	20 (22.2)	0	20 (33.3)
Common atrioventricular valve repair, *n* (%)	7 (7.7)	0	7 (11.6)
Infective endocarditis, *n* (%)	2 (2.2)	1 (3.3)	1 (1.6)
Rheumatic disease, *n* (%)	3 (3.3)	1 (3.3)	2 (3.3)

Abbreviation: IQR, interquartile range.

### Systemic semilunar valve repair

The characteristics of patients who underwent systemic semilunar valve repair are detailed in **Table 1**. The majority of patients, 63%, were male. The median age was 13 years (IQR, 6.9-16.7). Notably, the indication for repair for 87% of patients was primarily more than moderate regurgitation, whereas 13% had also more than moderate stenosis. The semilunar valves were unicuspid (3%), bicuspid (40%), or originally a truncal valve (16%) and the rest were tricuspid. A total of 9 (30%) patients had previous cardiac interventions including balloon valvuloplasty and truncal valve repair surgery.

### AV valve repair

The characteristics of patients who underwent AV valve repair are detailed in **Table 1**. A total of 24 (40%) of the patients were male, and 15 patients (25%) were under the age of 1 year. Left AV valve repairs were performed in 16 patients. A significant proportion of patients (80%) had a history of prior cardiac interventions, and 38% had a single ventricle physiology. The underlying conditions for single ventricle were hypoplastic left heart syndrome (HLHS; *n* = 11), unbalanced atrioventricular septal defect (AVSD; *n* = 8), tricuspid atresia (*n* = 5), isolated heterotaxy (*n* = 4), and Shone’s complex (*n* = 3). The previous cardiac procedures included balloon valvuloplasty, commissuroplasty, and common AV valve repair.

### Surgical techniques

#### Semilunar valve repair

The operative details and surgical repair techniques are provided in **Table 2**. Techniques for aortic stenosis management encompassed leaflet shaving and commissurotomy, while cusp prolapses were addressed through plication techniques or triangular resections of the cusp free edges. Root reimplantation techniques were employed in 3 patients. Bicuspid valve corrections involved closing the gap, prolonging the raphe between fused leaflets, and partial annuloplasty or reimplantation to create symmetric valves. Patch augmentation was used in five patients for leaflet repair. A total of 17 (56.7%) patients had complex valve repair. The distribution of the applied surgical techniques is visualized in **Figure S1**.

**Table 2.** Operative Details: Semilunar Valve Repair

Variable	Value
CPB time, min (IQR)	163 (106-187)
Cross clamp time, min (IQR)	104 (74-143)
Plication, *n* (%)	24 (80)
Triangular resection, *n* (%)	5 (16.6)
Patch augmentation, *n* (%)	5 (16.6)
Leaflet shaving, *n* (%)	11 (36.6)
Commissurotomy, *n* (%)	12 (40)
Subcommissural annuloplasty, *n* (%)	7 (23.3)
Raphe closure, *n* (%)	2 (6.6)
Partial annuloplasty, *n* (%)	5 (16.6)
Root reimplantation, *n* (%)	3 (10)
Complex valve repair, *n* (%)	17 (56.7)

Abbreviations: CPB, cardiopulmonary bypass; IQR, interquartile range.

#### AV valve repair

The operative details and surgical repair techniques are provided in **Table 3**. There were 20 tricuspid valve repairs (single ventricle: 13 and 2 ventricles: 7), with 8 patients undergoing patch augmentation in the anterior leaflet. Neochordae reconstruction (*n* = 7), commissuroplasty (*n* = 13), chordae resection (*n* = 1), ring annuloplasty (*n* = 5), and annuloplasty without a ring (*n* = 4) were performed. Fifteen patients underwent mitral valve repairs. For mitral regurgitation, 6 patients underwent neochordae reconstruction, while 3 patients received patch augmentation and annuloplasty including 1 patient for infective endocarditis. Secondary chordae resection (*n* = 1) and commissuroplasty (*n* = 4) were performed for mitral stenosis. Common AV valve repair was performed on 7 patients with single ventricle, applying techniques such as neochordae reconstruction (*n* = 4), annuloplasty (*n* = 4), bridging (*n* = 5), commissuroplasty (*n* = 3), and cleft closure (*n* = 5). A total of 28 (46.7%) patients had complex valve repair. The distribution of the applied surgical techniques is visualized in **Figure S2**.

**Table 3.** Operative Details: Atrioventricular Valve Repair

Variable	All AV valve repair (*n* = 60)	Mitral valve repair (*n* = 15)	Tricuspid valve repair (*n* = 20)	Common AV valve repair (*n* = 7)
CPB time, min (IQR)	141 (113-202)	—	—	—
Cross clamp time, min (IQR)	90 (70-121)	—	—	—
Neochordae implantation, *n* (%)	31 (51.6)	6 (10)	7 (11.6)	4 (6.6)
Commissuroplasty, *n* (%)	23 (38.3)	4 (6.6)	13 (21.6)	3 (5)
Patch augmentation of the leaflet, *n* (%)	13 (21.6)	3 (5)	8 (13.3)	0
Chordae resection, *n* (%)	3 (5)	1 (1.6)	1 (1.6)	0
Annuloplasty without ring, *n* (%)	23 (38.3)	4 (6.6)	9 (15)	4 (6.6)
Annuloplasty with ring, *n* (%)	8 (13.3)	3 (5)	5 (5)	0
Cleft closure without patch, *n* (%)	12 (20)	0	2 (3.3)	5 (5)
Cleft closure with patch, *n* (%)	12 (20)	0	0	1 (1.6)
Edge to edge, *n* (%)	1 (1.6)	0	0	
Bridging, *n* (%)	5 (8.3)	0	0	5 (5)
Leaflet shaving, *n* (%)	7 (11.6)	6 (10)	0	1 (1.6)
Commissurotomy, *n* (%)	7 (11.6)	7 (11.6)	0	0
Papillary muscle splitting, *n* (%)	6 (10)	6 (10)	0	0
Complex valve repair, *n* (%)	28 (46.7)	9 (15)	9 (15)	6 (10)

Abbreviations: AV, atrioventricular; CPB, cardiopulmonary bypass; IQR, interquartile range.

### Clinical outcomes

The median duration of the follow-up period was 7 months (IQR, 1.5-21.4) (**Table 4**). A total of 9 (10%) patients died during this follow-up period; 4 (4.4%) died within 30 days after surgery and 5 (5.6%) after a median of 235 days (IQR, 127-343). Among these 9 patients, 6 had hypoplastic left heart syndrome (HLHS) or an HLHS variant. The causes of death were cancer (*n* = 1), multiple organ failure (*n* = 3), and hypoxic brain injury (*n* = 2).

**Table 4.** Clinical Outcomes

Follow-up periods, month (IQR)	7 (1.5-21.4)
Mortality, *n* (%)	9 (10)
Early mortality, *n* (%)	4 (4.4)
Late mortality, *n* (%)	5 (5.6)
Reoperation for the valve, *n* (%)	4 (4.4)
Early reoperation, *n* (%)	2 (2.2)
Late reoperation, *n* (%)	2 (2.2)
Valve replacement, *n* (%)	4 (4.4)
Deterioration/Failure, *n* (%)	4 (4.4)

Abbreviation: IQR, interquartile range.

**Figure 1.**
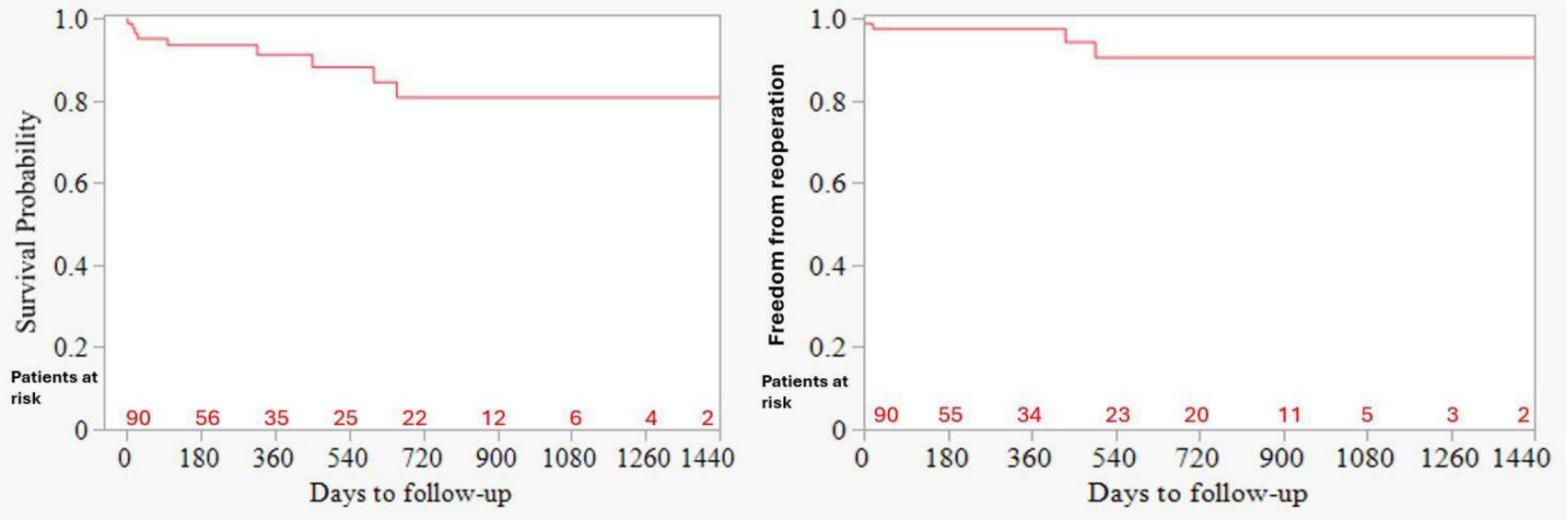
Estimated Survival (Left) and Freedom from Reoperation (Right) for the Whole Cohort

**Figure 2.**
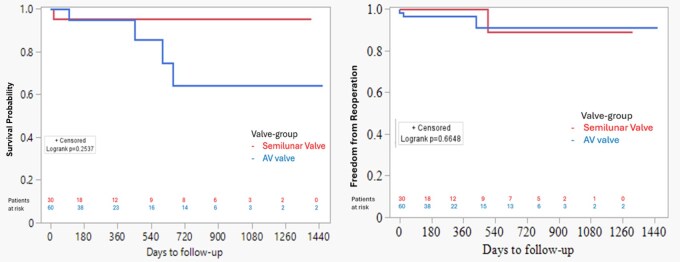
Estimated Survival (Left) and Freedom from Reoperation (Right) for Each Group

Kaplan-Meier analysis revealed that estimated survival for whole patient population was 93.6% at 6 months and 91.3% at 12 months (**Figure 1**). Estimated survival at 6 months for the semilunar and AV valve groups were 95.2% and 95%, respectively. Estimated survival at 12 months for semilunar and AV valve groups were 95.2% and 95%, respectively. There was no significant difference in estimated mortality between both groups (*P* = .25) (**Figure 2**).

A total of 4 (4.4%) patients had reoperation with 2 (2.2%) being early and 2 (2.2%) late reoperations. The estimated freedom from reoperation of the valve that was originally repaired in whole patient population was 97.5% at 6 months and 94.3% at 12 months (**Figure 1**). The estimated freedom from reoperation at 6 months for repair of semilunar and AV valves were 95.8% and 95.6%, respectively. Estimated freedom from reoperation at 12 months for repair of semilunar and AV valves were 95.8% and 95.6%, respectively. There was no significant difference in estimated freedom from reoperation between both groups (*P* = .66) (**Figure 2**).

On bivariable analysis single ventricle morphology (hazard ratio [HR], 5.2, 95% confidence interval [CI], 1.3-20.8; *P* = .0198) was associated with increased risk of all-cause mortality. Older age at time of surgery was found to be predictor of decreased risk of all-cause mortality (HR, 0.7, 95% CI, 0.6-0.9; *P* = .0223). Weight at time of surgery (*P* = .811), complexity of semilunar repair (*P* = .218), complexity of AV valve repair (*P* = .972), and bicuspid aortic valve (*P* = .888) were not associated with all-cause mortality. Due to limited sample size, we were not able to assess the predictors for reoperation.

### Reoperations and repair failure

A total of 2 patients necessitated redo valve repairs. One patient underwent common AV valve repair using bridging technique, during the interstage period between the Glenn and Fontan procedures. The common AV valve regurgitation had increased level by the time of the Fontan surgery. During the Fontan procedure, it was discovered that the Gore-Tex bridge had become detached from the valve. Another patient required redo common AV valve repair 1-month post-surgery due to a ruptured implanted neochordae.

One patient with Shone’s syndrome with aortic stenosis and combined mitral disease underwent aortic and mitral valve repair at 35 days of age. After initial recovery, the patient needed prolonged stay at intensive care unit due to respiratory insufficiency without being weaned from invasive ventilation. This situation necessitated a redo operation in the following month for severe mitral insufficiency. Intraoperative findings, necrotic papillary muscles, led to failing of another repair of the valve so that the valve had to be replaced with a mechanical prothesis.

Another patient, 4 months of age, underwent mitral valve repair and anomalous left coronary artery from the pulmonary artery (ALCAPA) repair. Two weeks postoperatively, the patient suffered from cardiac arrest with need of extra-corporeal membrane oxygenation. Due to severe mitral regurgitation the patient had to be reoperated. A severely dysplastic valve left no choice but to replace the mitral valve with a mechanical prothesis.

Additionally, more than moderate regurgitation was observed in 4 patients following surgery. Among these cases, 1 patient exhibited ruptured chordae, while another manifested severe aortic regurgitation subsequent to an initial surgery for congenital aortic valve stenosis. A severe myocarditis case that mandated ventricular assist device implantation at 15 days of age resulted in persistent severe aortic regurgitation, which necessitated subcommissural annuloplasty at the time of the repair. One patient, who had an HLHS variant, displayed severe right AV regurgitation following the Norwood procedure. In response, an approach involving neochordae reconstruction and commissuroplasty was undertaken, resulting in an improvement from severe to moderate valve regurgitation.

Four patients, all of whom presented with an HLHS variant, demonstrated severe right AV regurgitation subsequent to the Norwood procedure. To address this, neochordae reconstruction and commissuroplasty were performed, yielding only a decrease from severe to moderate valve regurgitation.

## DISCUSSION

Valve repair procedures are common in paediatric populations, offering substantial benefits over valve replacement, especially concerning anticoagulation, ventricular function, and the potential for somatic growth.^5^ There is now growing evidence that implantation of a mechanical or bioprosthetic valve in the aortic position decrease survival when compared to the Ross procedure and valve repair.^6^ Additionally, there are data that suggest mitral valve replacement is associated with higher mortality and transplant rates in comparison to mitral valve repair.^7^ Our surgical experience encompasses a diverse array of patients, including complex cases such as common AV valve repairs in small children. Notably, 40% of AV valve repairs were performed on patients with a single ventricle. We are now realizing that a large proportion of the patients with dominant right single ventricle necessitate repair over the first decades of life.^8^^,^^9^ Given the diversity of valve repair procedures we performed, a comprehensive discussion of outcomes becomes challenging. Mortality rates were heavily influenced by individual patient conditions, especially in patients with HLHS.

It is crucial to acknowledge that valve repair techniques in paediatric patients are frequently more intricate and less readily standardized compared to their adult counterparts due to the presence of complex and often dysplastic morphologies.^10^ Consequently, a diverse range of surgical techniques is often necessitated within the paediatric population. In this specific cohort, it is noteworthy that the most senior surgeon was responsible for the majority of surgeries, and they performed a higher number of complex repairs using combination of three or more techniques compared to the other surgeons. As valve repair becomes a fundamental part of our armamentarium in paediatric cardiac surgeons, we should focus on organizing teaching of the younger generations of surgeons.

### Semilunar valve repair

The types of diseases necessitating aortic valve surgeries vary across age groups. In neonates and infants, congenital aortic stenosis predominates as the primary indication for aortic valve repair. Repair of congenital aortic valve stenosis can provide replacement free long-term results.^11^ Techniques of surgical repair for this type have evolved and always include an extensive debridement of the valve by resection of all nodular dysplasia, thinning of the leaflets, and recreation of an interleaflet triangle by resecting the fibrosis immobilizing the commissure. Incised portions of stenotic valves are resuspended with pericardial patches to avoid regurgitation.^12^ In teenagers, we conducted valve repairs for bicuspid valves or valve regurgitation arising from root enlargement. For bicuspid valves, we restored cusp configuration and increased the effective height through cusp plication, resection, or raphe closure. In cases of root dilation, we employed the reimplantation technique in 3 patients. In situations where commissure orientation was asymmetrical, partial annuloplasty (*n* = 5), or root reimplantation techniques were used to achieve symmetrical orientation.^13^

### AV valve repair

Mitral valve repair, especially when performed at older age, can yield reliable long-term results in children in terms of mortality and reoperation rates.^14^ Among the 60 patients who underwent AV valve repair, 38.3% had a single ventricle morphology. Patients with single ventricle physiology and common AV valve pathology are known to experience a continuous decline in valve function, with the majority encountering valve failure within the first 30 years of life.^8^ Despite employing volume-unloading staged procedures and various valve repair techniques, AV valve repair remains a significant long-term mortality risk factor after the Fontan procedure.^15^^,^^16^ Our approach to AV valve repair was tailored to the mechanism of regurgitation. For common AV valve regurgitation, we employed the polytetrafluoroethylene (PTFE) bridge technique.^17^ A 0.4-mm PTFE patch was trimmed and secured to the anterior and posterior valve annulus with CV-6 PTFE (Gore-Tex) sutures. The PTFE strip was secured to the leaflet with several separate polypropylene sutures. We also conducted tricuspid valve repair in patients with single ventricle. For tricuspid valve repair, we augmented anterior leaflet with bovine pericardium patch if the patient has functional regurgitation (*n* = 5).

Left AV valve regurgitation after AVSD repair was common cause of redo AV valve repair (*n* = 16, 26.6%). It is the main indication for reoperation in patients after repair of both partial and complete AVSD.^18^ Typically, regurgitation results from inadequate coaptation following primary cleft closure In these cases, we typically augment the cleft with a patch.^19^ The patch was trimmed and sutured to the edge of the leaflet, with neochordae attached to the patch and neighbouring marginal chordae.

### Limitations

There are several limitations of this study that should be noted. First, this is a single-centre retrospective study with the corresponding limitations associated with its nature. Second, the number of patients included in the study is limited due to the relatively small time frame. Consequently, these data could not be stratified by age groups for meaningful analysis. Additionally, we could only obtain short-term outcome data. A limitation of our study is the use of complete-case analysis for handling missing data; although the proportion of missingness was small, this approach may still introduce bias if the data were not missing completely at random.

## CONCLUSION

Valve repair in congenital heart surgery practice in our centre reflects the dynamic nature of paediatric cardiac surgery and our early outcomes have demonstrated the benefits it offers to our patients. Ongoing research will be essential in further refining and optimizing valve repair strategies for the diverse population of congenital heart disease patients.

## AUTHOR CONRIBUTIONS

Muhammed Ikbal Aydin (Conceptualization; Data curation; Formal analysis; Investigation; Methodology; Project administration; Supervision; Writing—original draft), Eiri Kisamori (Conceptualization; Data curation; Formal analysis; Investigation; Methodology; Supervision; Writing—original draft), Mitchell Haverty (Data curation; Investigation; Validation), Rittal Mehta (Data curation; Formal analysis), Aybala Tongut (Supervision; Writing—review & editing), Manan Desai (Supervision; Writing—review & editing), Gerard Martin (Supervision; Writing—review & editing), Wayne Jay Franklin (Supervision; Writing—review & editing), and Yves d’Udekem (Conceptualization; Investigation; Methodology; Project administration; Supervision; Writing—review & editing)

## Supplementary Material

ivaf273_Supplementary_Data

## Data Availability

Derived data supporting the findings of the study are available upon reasonable request from the corresponding author.
