# Supplementary material for: Early Favourable Outcomes of Valve Repair in Congenital Heart Surgery
Source: Interdiscip Cardiovasc Thorac Surg. 2025 Nov 26;40(12):ivaf273. doi: 10.1093/icvts/ivaf273 (PMC12782726; doi:10.1093/icvts/ivaf273)
Supplement: ivaf273_Supplementary_Data [file ivaf273_supplementary_data.zip › Supplementary Figures 1-2.docx]

Supplementary Figure 1

Supplementary Figure 2
